# Awareness Level About Cervical Cancer, Human Papillomavirus (HPV) and Corresponding Vaccine Among Women Living in the Western Region of Saudi Arabia

**DOI:** 10.7759/cureus.37512

**Published:** 2023-04-12

**Authors:** Raghad O Alkhaldi, Huda A Alzahrani, Lobna A Metwally

**Affiliations:** 1 Medicine and Surgery, Taif University, Taif, SAU; 2 Medicine, Taif University, Taif, SAU

**Keywords:** saudi arabia, human papillomavirus, hpv vaccine, cervical cancer, hpv infection

## Abstract

Background

In this study, we aimed to evaluate the knowledge and awareness about cervical cancer, human papillomavirus (HPV) and its vaccine among women living in the western region of Saudi Arabia.

Methodology

Using a cross-sectional online survey, this study assesses the knowledge and awareness level of HPV and the risk factors of cervical cancer among women living in the western region of Saudi Arabia. The design of the questionnaire is based on several earlier studies in different populations.

Results

The total completed responses include a sample of 624 that was subjected to statistical analysis; the analysis showed that only 34.6% were aware of HPV. Participants who belonged to the 21-30 and 31-40-year-old groups had comparatively more awareness than other age groups (p<0.001). Most (83.8%) believed that it would cause cervical cancer. Less than half (45.8%) knew that there is a vaccine against HPV infection. When we assessed the willingness to receive the vaccine, it was found that 75.8% were willing to take it.

Conclusions

The study found that women in the western region of Saudi Arabia have limited knowledge of cervical cancer and HPV and its vaccine. There is a need to educate and promote awareness of HPV and its complications for women in the western region of Saudi Arabia.

## Introduction

Human papillomavirus (HPV) is considered a sexually transmitted virus and is strongly linked to many types of cancer, such as cervical, ovarian, and vaginal [1]. HPV has a strong resistance to heat and dryness and nonsexual transmission through fomites and exposure to shared contaminated clothing for a long time [2]. Two classes of HPV exist: high-risk (HR-HPV) and low-risk. This classification is based on their oncogenic inducing capabilities. The high-risk group, which currently includes HPV 16, 18, 31, 33, 35, 39, 45, 51, 52, 56, 58, 59, 68, 73, and 82, has been related to cervical intraepithelial lesions and may progress into cervical cancer [3].

The upper cervix (endocervix) is lined with a simple columnar epithelium, and the lower cervix (ectocervix) is lined with stratified squamous epithelium. At the junction between them, there is a high chance of the occurrence of metaplastic changes; therefore, most of the cervical cancers arise from within this site. The hazard of HPV infection corresponds to the highest levels of metaplastic activity. The highest metaplastic activity occurs in puberty and the first pregnancy and decreases after menopause [4].

The most prevalent cancer that affects women’s health is cervical cancer, which is the fourth most prevalent cancer among women in the world. According to the World Health Organization (WHO), cervical cancer is the second most prevalent cancer among women residing in less developed regions, with a reported 604,000 new cases in 2020. Approximately 342,000 cases died from cervical cancer in 2020, about 90% of these deaths occurring in less developed regions [5].

In Saudi Arabia, around 6.5 million women over 15 years old are at risk of developing cervical cancer, according to the World Health Organization [6]. Cervical cancer is the ninth most commonly diagnosed cancer in women aged 15-44 years old in the Kingdom of Saudi Arabia (KSA) [7]. The estimated incidence of cervical cancer in Saudi women, according to the GLOBOCAN report of 2012, is about 2.2 per 100,000 age-standardized rate (ASR), and 84 (34.8%) of 241 women have died due to cervical cancer in 2012 [8]. The pap smear to detect premalignant lesions and the availability of vaccines are ways to prevent cervical cancer, which could also be curable by detecting early warning signs and symptoms. Most women in developing countries, including KSA, require extensive treatment modalities like surgery, radiotherapy, and chemotherapy because they are presented at an advanced stage [9].

Consistent evidence shows that the authorized bivalent and quadrivalent HPV vaccines containing HPV16 and 18 antigens defend against HPV infection, and precancerous cervical lesions correlate with these types while individuals are not yet exposed [10]. For the last 10 years, the HPV vaccine has been provided in the KSA and is accessible in large hospitals with or without monetary charges [11]. In KSA, the uptake of the HPV vaccine is still low [12,13], which is attributed to a variety of reasons, including an inadequate understanding of HPV and its connection to cervical cancer, a lack of recognition of the HPV vaccination, and the belief that HPV is a sensitive topic in the conservative Saudi community [14,15].

In this study, we tried to assess the knowledge and awareness level about cervical cancer, the mucosal type of HPV and its vaccine among women living in the western region of Saudi Arabia.

## Materials and methods

Study design

A quantitative and descriptive cross-sectional study was done.

Study participants

The study targeted every female aged between 18 and 50 years in the western region of Saudi Arabia. The exclusion criteria were men and women living in the eastern, middle, south, and northern regions of Saudi Arabia.

Study instruments

A pre-designed online questionnaire was used. The questionnaire was distributed randomly via social media and delivered to (703) women during the period from September 2020 to January 2021. We invited them to participate in the survey, and the purpose of the study was explained to them before sharing in the study. The questionnaire consisted of four parts and covered the data about participants' socio-demographics (age, education level, residency, and social status). In addition, it contained questions about participants' awareness about cervical cancer, papillomavirus (HPV) (“true,” “false,” and “do not know”), and acceptance of HPV vaccination (“yes,” “no”).

Data analysis

The data were analyzed by the SPSS program version 23 (SPSS Inc., Armonk, NY). Qualitative data are expressed as a number and percentage, and quantitative data are expressed as the mean and standard deviation (SD). A suitable statistical test will be used accordingly. P-values <0.05 are considered significant.

Ethical considerations

This study obtained ethical approval from the Research Ethical Committee at Taif University, KSA (No. 42-0017). The questionnaires were anonymous, ensuring confidentiality.

## Results

This study was done to assess the awareness and knowledge about human papillomavirus (HPV) among the adult female population residing in the western region of Saudi Arabia. The total completed responses include a sample of 624 that was subjected to statistical analysis. The age distribution of the participants showed that 29.6% were 18-20 years old, 48.4% were 21-30 years old, and 13.1% were 31-40 years old. The majority of the participants (83.7%) had educational levels at the graduate level or more, and 73.6% of the participants were single (Table 1).

**Table 1:** Socio-demographic details of the participants.

		Frequency	Percent
Age	18-20	185	29.6
	20-30	302	48.4
	30-40	82	13.1
	40-50	44	7.1
	>50	11	1.8
Educational level	Primary	1	0.2
	Secondary	2	0.3
	Middle	99	15.9
	Graduate	522	83.7
Marital status	Single	459	73.6
	Married	150	24.0
	Divorced	14	2.2
	Widow	1	0.2

The analysis showed that only 34.6% (n=216) were aware of HPV, and hence, the assessment of knowledge related to HPV and associated factors were done only among these participants. When we assessed the relationship of the level of awareness with the participants’ different socio-demographic characteristics, there was no statistically significant association seen except for age. The 21-30-year-old and 31-40-year-old participants had comparatively more awareness than the other age groups (p<0.001) (Table 2).

**Table 2:** Awareness and socio-demographic characteristics (n=624). *A p-value less than 0.05 is considered statistically significant. HPV: human papillomavirus.

			Awareness of HPV		Total	P-value*
			Aware	Not aware		
Age (in years)	18-20	N	46	139	185	<0.001
		%	24.9	75.1	29.6	
	21-30	N	134	168	302	
		%	44.4	55.6	48.4	
	31-40	N	33	49	82	
		%	40.2	59.8	13.1	
	41-50	N	2	42	44	
		%	4.5	95.5	7.1	
	>50	N	1	10	11	
		%	9.1	90.9	1.8	
Educational level	Primary	N	1	0	1	0.538
		%	100.0	0.0	0.2	
	Middle	N	33	66	99	
		%	33.3	66.7	15.9	
	Secondary	N	1	1	2	
		%	50.0	50.0	0.3	
	Graduate	N	181	341	522	
		%	34.7	65.3	83.7	
Marital status	Single	N	162	297	459	0.683
		%	35.3	64.7	73.6	
	Married	N	48	102	150	
		%	32.0	68.0	24.0	
	Divorced	N	6	8	14	
		%	42.9	57.1	2.2	
	Widow	N	0	1	1	
		%	0.0	100.0	0.2	

When the relationship between these levels of knowledge and various socio-demographic details was evaluated, it was found that participants who belonged to 21-30 years and 31-40 years comparatively had more "good" knowledge than others (p=0.042). There was no statistically significant association observed with the participants’ educational level and marital status (Table 3).

**Table 3:** Knowledge level and socio-demographic characteristics (n=216). *A p-value less than 0.05 is considered statistically significant.

			Total class			Total	P-value*
			Good	Fair	Poor		
Age (in years)	18-20	N	21	12	13	46	0.042
		%	45.7	26.1	28.3	21.3	
	21-30	N	47	32	55	134	
		%	35.1	23.9	41.0	62.0	
	31-40	N	6	6	21	33	
		%	18.2	18.2	63.6	15.3	
	41-50	N	2	0	0	2	
		%	100.0	0.0	0.0	0.9	
	>50	N	1	0	0	1	
		%	100.0	0.0	0.0	0.5	
Educational level	Primary	N	0	0	1	1	0.251
		%	0.0	0.0	100.0	0.5	
	Secondary	N	17	4	12	33	
		%	51.5	12.1	36.4	15.3	
	Middle	N	0	0	1	1	
		%	0.0	0.0	100.0	0.5	
	Graduate or more	N	60	46	75	181	
		%	33.1	25.4	41.4	83.8	
Marital status	Single	N	60	38	64	162	0.319
		%	37.0	23.5	39.5	75.0	
	Married	N	13	11	24	48	
		%	27.1	22.9	50.0	22.2	
	Divorced	N	4	1	1	6	
		%	66.7	16.7	16.7	2.8	

When we assessed the willingness to receive the vaccine for HPV, it was found that 75.8% were willing to take it. In participants who were not willing to receive the HPV vaccine (n=151), 30.5% mentioned that they are against vaccines, 32.5% believed that this vaccine is not necessary, and 15.9% reported that they are unsafe (Table 4).

**Table 4:** Willingness to receive vaccine and reasons for not accepting it. HPV: human papillomavirus.

	N	%
Willingness to receive HPV vaccine (n=624)		
Yes	473	75.8
No	151	24.2
Reasons for not taking HPV vaccine (n=151)		
I am against vaccines in general	46	30.5
The HPV vaccine is not necessary	49	32.5
The vaccine is not safe	24	15.9
Other	25	16.6
No reason	7	4.6

When we assessed the relationship of willingness based on the knowledge level, it was found that 94.8% of the participants who had "good" knowledge were willing to take the vaccine, which showed a statistically significant relationship (p=0.001) (Table 5).

**Table 5:** Willingness to receive the vaccine and its relationship with the level of knowledge (n=216).

			Willingness to receive vaccine		Total	P-value
			Yes	No		
Knowledge level	Good	N	73	4	77	0.001
		%	94.8	5.2	35.6	
	Fair	N	47	3	50	
		%	94.0	6.0	23.1	
	Poor	N	69	20	89	
		%	77.5	22.5	41.2	

## Discussion

The present study explored the awareness and knowledge regarding human papillomavirus (HPV) infection and willingness to accept vaccines for it among adult females in the western region of Saudi Arabia. We found that the awareness about HPV in these females was significantly less (34.6%), and out of this, approximately one-third had only good knowledge regarding the infection and the risks involved. A recent study conducted in four Arab countries (Iraq, Jordan, Qatar, and the United Arab Emirates) has reported poor awareness (26.1%) compared to our study findings [16]. In another study done in Nepal, women reported very poor awareness (15.4%) of HPV [17]. In our study, the majority of the females agreed that HPV is a sexually transmitted disease. This percentage is higher compared to the findings of another study done among adult women attending primary care clinics in Riyadh, which reported that only 32.5% knew that this disease could be sexually transmitted, and 21.5% believed this disease has a vaccine [8].

According to the WHO’s health statistics report, females aged between 35 and 44 years are at an increased risk of getting cervical cancer [18]. In this study, the majority of females believed that HPV could cause cervical cancer. It is estimated that about 10% of long-standing HPV infection (high-risk HPV) has a very high risk for progression to cervical cancer [19,20]. It is well known that screening for cervical cancer drastically reduces the incidence and mortality associated with it [21,22]. But the utilization of screening for cervical cancer is lower compared to other types of cancer [21-24]. A clinical trial conducted in India showed that even once-a-lifetime screening for cervical cancer significantly reduced the incidence of advanced cervical cancer and the mortality rates compared to no screening [25]. It was interesting to find that despite the low awareness regarding HPV, more than two-thirds of the participants were willing to receive the vaccine. According to the Center for Disease Control's (CDC) guidelines, it is recommended to give the HPV vaccine to both girls and boys aged between 11 and 12 years [26]. In Saudi Arabia, two of the above vaccines started to available from the year, and efforts are being made to increase their distribution [27]. It was reported that women who were aware of the relationship of HPV infection with cervical cancer were ten times more likely to take the vaccine [17,28]. According to the Saudi Cancer Registry report, cervical cancer incidence is very low in women (2.1/100,000) [13]. Although vaccination is the best effective measure to reduce the incidence of many diseases, there is no logic for public health organizations to implement either screening or vaccination for HPV if the country has a very low incidence. It may also be argued that implementing such preventive programs may not sensibly reduce the incidence at the national level, as it is already very low. When vaccinating, it is always better to target young females as they have higher immunogenicity than others. Our study found that the most common reason for not taking the HPV vaccine was because they are against it. A study conducted in California among those who accepted and refused to take the HPV vaccine reported that the most common reported reason to accept the vaccine was their physician’s recommendation to take it. In comparison, the main reason to refuse the vaccine was the need for further research or information on the vaccine’s efficacy and safety [29].

This study finding shows that there is a need to promote awareness and educate Saudi women regarding HPV and its complications. Even though vaccination is an effective way of preventing infection, other preventive strategies, such as lifestyle modifications, proper sex education, abstinence from sexual intercourse, and the use of barrier contraception methods should also be emphasized when educating the public. Healthcare professionals have a crucial role to play in educating and motivating patients to institute and achieve HPV vaccination. An annual national HPV awareness campaign could be organized, targeting both parents and young females.

## Conclusions

This study set out to assess the evaluate the knowledge and awareness about cervical cancer, human papillomavirus (HPV) and its vaccine among women living in the western region of Saudi Arabia. This study has identified that women in the western region of Saudi Arabia have limited knowledge of cervical cancer and HPV and its vaccine. Therefore, it is crucial to increase women's awareness of cervical cancer and HPV and the importance of its vaccine through an awareness campaign.
